# Double burden of COVID-19 knowledge deficit: low health literacy and high information avoidance

**DOI:** 10.1186/s13104-022-05913-8

**Published:** 2022-02-05

**Authors:** Xuewei Chen, Ming Li, Gary L. Kreps

**Affiliations:** 1 School of Community Health Sciences, Counseling and Counseling Psychology, Oklahoma State University, 429 Willard Hall, Stillwater, OK 74078 USA; 2 Department of Health Sciences, College of Health Professions, Towson University, 8000 York Road, Linthicum Hall, Room 101B, Towson, MD 21252 USA; 3 Department of Communication, Center for Health and Risk Communication, George Mason University, 4400 University Drive, Fairfax, VA 22030 USA

**Keywords:** Health literacy, Information avoidance, COVID-19

## Abstract

**Objective:**

People with lower levels of health literacy are likely to report engaging in information avoidance. However, health information avoidance has been overlooked in previous research on responses to viral outbreaks. The purpose of this cross-sectional survey study was to assess the relationship between health literacy and COVID-19 information avoidance. Students (n = 561) at a university in the south central region of the U.S. completed our online survey conducted from April to June 2020 using simple random sampling. We measured information avoidance and the degree to which people opt not to learn about COVID-19 when given the choice. We assessed participants’ health literacy level using the Newest Vital Sign (NVS), eHealth Literacy Scale (eHEALS), and All Aspect of Health Literacy Scale (AAHLS).

**Results:**

Those with lower health literacy were more likely to avoid information about COVID-19. This negative association between health literacy and information avoidance was consistent across all types of health literacy measures: NVS scores (b = − 0.47, p = 0.033), eHEALS scores (b = − 0.12, p = 0.003), functional health literacy (b = − 0.66, p = 0.001), communicative health literacy (b = − 0.94, p < 0.001), information appraisal (b = − 0.36, p = 0.004), and empowerment (b = − 0.62, p = 0.027). The double burden of low health literacy and high information avoidance is likely to lead to a lack of knowledge about COVID-19.

## Introduction

Information avoidance is defined as “any behavior intended to prevent or delay the acquisition of available but potentially unwanted information” [1]. The reasons for avoiding attending to health information include holding on to previous beliefs, reducing undesired actions, and decreasing unwanted emotions [1]. People with higher health information avoidance tendencies are less likely to engage in protective health behaviors [2]. Moreover, those with higher tendencies to avoid attending to COVID-19 related health information are less likely to perform behaviors such as physical distancing and wearing a mask to prevent against the virus [3, 4]. People with lower levels of health literacy are likely to report engaging in information avoidance [5, 6]. Researchers found that lower health literacy is associated with more avoidance of COVID-19 information among community participants in Germany [4]. Our current study examines whether this occurs in the United States as well.

We identified two issues in the current literature. First, health information avoidance has often been overlooked in previous research on responses to viral outbreaks [4]. To address this issue, this study aimed to assess individuals’ health information avoidance related to COVID-19 and examine the relationship between health literacy and COVID-19 information avoidance through a cross-sectional online survey study among college students at a public land-grant university in the south central region of the U.S. Another issue is related to the measurement of health literacy. Health literacy is a multidimensional concept [7]. There are more than 51 measures available to assess a person’s health literacy, but they generally represent a narrow set of conceptual dimensions [8]. A prior study pointed out that self-reported, perception based health literacy and objective, performance-based health literacy should be treated as separate concepts [9]. To address the health literacy measurement issue, we applied multiple health literacy measures assessing both objective and subjective health literacy covering various dimensions.

## Main text

### Methods

#### Participants and data collection procedure

We collected data for this cross-sectional online survey study among students at XX University [blind for peer review], a public land-grant university in the south central region of the U.S., between April and June 2020. During the data collection period, the total COVID-19 cases in the XX state [blind for peer review] increased from around 300 to 11,000 [10]. Study recruitment flyers were distributed to randomly selected university-system student e-mail addresses by University Institute for Research and Information Management using simple random sampling strategy. Those who were interested in participating used the anonymous link or QR code to take our Qualtrics survey. Before starting the survey, the screen presented our study consent form and participation criteria. If the potential participants confirmed that they met the participation criteria and electronically signed the study consent form, they would continue to the survey. Our participation criteria included being (a) a student enrolled at XX University [blind for peer review], (b) 18 years or older, (c) proficient in English, and (d) physically located in the United States. The first 120 participants received a $5 Amazon electronic gift card as incentives. We included a final sample size of 561 in our data analysis because these participants completed the survey with valid responses (i.e., passing both of the survey validation items). Based on rule-of-thumb for multiple regression with seven predictors: N ≥ 50 + 8p (where p is the number of predictors) [11], sample size larger than 106 should be sufficient. Also, based on power analysis, our 561 participants would provide 100% power to detect a good effect size in a regression model with seven predictors.

#### Measures (see detailed descriptions of each measure in Table 1)

**Table 1:** Description, examples, and data reliability of the measures in the survey

Measures	# of items	Example question	Theoretical range	Actual range	Mean	SD	Cronbach’s alpha	Interpretation
NVS	6	If you eat the entire container, how many calories will you eat?	0–6	0–6	5.22	1.14	0.60	Higher score = higher level of health literacy
eHEALS	8	I know how to find helpful health information resources on the Internet [1 = Strongly disagree; 5 = Strongly agree]	8–40	10–40	32.04	6.01	0.90	Higher score = higher level of health literacy
AAHLS							0.70	Higher score = higher level of health literacy
AAHLS-functional health literacy	3	Do you need help to fill in official documents? [1 = often; 3 = rarely]	3–9	4–9	7.68	1.17		
AAHLS-communicative health literacy	3	When you talk to a doctor or nurse, do you make sure they explain anything that you do not understand? [1 = rarely; 3 = often]	3–9	3–9	7.91	1.40		
AAHLS-information appraisal	4	Are you someone who likes to find out lots of different information about your health? [1 = rarely; 3 = often]	4–12	4–12	9.12	1.98		
AAHLS-empowerment	3	Do you think that there are plenty of ways to have a say in what the government does about health? [1 = yes; 0 = no]	0–3	0–3	1.77	0.85		
Information Avoidance	7	Even if it will upset me, I want to know about COVID-19. [1 = Strongly agree; 5 = Strongly disagree]	7–35	7–35	12.34	5.77	0.90	Higher score = higher level of information avoidance

##### Health literacy

We assessed participants’ health literacy level using one objective test: the Newest Vital Sign (NVS) and two self-reported measures: eHealth Literacy Scale (eHEALS) and All Aspect of Health Literacy Scale (AAHLS). The NVS is an objective test asking participants to interpret a mock-up ice-cream nutrition label and answer six open-ended questions [12]. Answers to each question were scored either as correct (coded as 1) or incorrect (codes as 0). The eHEALS is a self-reported survey questionnaire to measure people’s perceived skills at using information technology for health [13]. The AAHLS is a self-reported survey that developed based on Nutbeams’ health literacy conceptual model assessing health literacy as three levels: functional health literacy (the ability to understand factual health information), communicative health literacy (the ability to act independently in a supportive environment), and critical health literacy (the ability to control health-related situations) [14]. Critical health literacy contains two components: information appraisal and empowerment [15].

##### Information avoidance

We measured information avoidance, the degree to which people opt not to learn about COVID-19 when given the choice, using seven items on a five-point Likert scale. These items were adapted from previous research [16, 17].

##### Covariates

Besides health literacy, health information avoidance is also associated with perceived risk and worry. Studies show that people are less likely to avoid health information if they have higher perceived risk and more worry about getting the disease [18–21]. Therefore, we included COVID-19 perceived risk and worry as covariates. On a five-point Likert scale, we asked participants to rate their perceived risk of getting COVID-19 in their lifetime (extremely unlikely to extremely likely) and how much they were worried about getting COVID-19 (not at all to extremely). Sociodemographic variables included sex, age, education (undergraduate or graduate), and race/ethnicity (White, Hispanic/Latino, Black or African American, American Indian or Alaska Native, Asian, and Other).

#### Data analysis

We performed two simple linear regression models to examine the associations between health information avoidance and perceived risk and worry about getting COVID-19. We then performed six sets of multiple linear regression models to clarify the relationship between health information avoidance and health literacy, controlling for demographic characteristics (i.e., sex, age, education, race/ethnicity) as well as perceived risk and worry. Each multiple linear regression model contained one health literacy measure: NVS, eHEALS, AAHLS functional health literacy, AAHLS communicative health literacy, AAHLS information appraisal, and AAHLS empowerment. We used Stata 16 for descriptive and regression analysis. The significance level was set at α = 0.05.

### Results

Participants’ age ranged from 18 to 65 (M = 24.99, SD = 7.47). More than half of them (50.54%) were between 18 and 22 years old. Less than 5% of the participants were aged above 40. Information about participants’ demographic characteristics and COVID-19 risk perceptions is listed in Table 2. More than half of the participants (50.26%) perceived that they were somewhat or extremely likely to get COVID-19 in their lifetimes. About 33.16% of the participants indicated that they were moderately or extremely worried about getting COVID-19. Generally, the participants had relatively high levels of health literacy and low levels of information avoidance.

**Table 2:** Participants’ demographic characteristics and COVID-19 perceptions (N = 561)

Demographics	n	%
Sex		
Male	203	36.19
Female	358	63.81
Race/ethnicity		
White	377	67.20
Hispanic/Latino	45	8.02
Black or African American	37	6.60
American Indian or Alaska Native	34	6.06
Asian	52	9.27
Other	16	2.85
Academic level		
Freshman	68	12.21
Sophomore	87	15.51
Junior	87	15.51
Senior	118	21.03
Master’s student	105	18.72
PhD student	56	9.98
Professional student (e.g., attending law school or medical school)	36	6.42
Missing	4	0.71
Perceived risk of getting COVID-19		
Extremely unlikely	41	7.31
Somewhat unlikely	109	19.41
Neither likely nor unlikely	129	22.99
Somewhat likely	215	38.32
Extremely likely	67	11.94
Worried about getting COVID-19		
Not at all	98	17.47
Slightly	143	25.49
Somewhat	134	23.89
Moderately	127	22.64
Extremely	59	10.52

#### Information avoidance and perceived risk and worry

The results of the simple linear regressions indicated that those with lower perceived risk of getting COVID-19 (b = − 0.88, p < 0.001) and who were less worried about COVID-19 (b = − 1.16, p < 0.001) were more likely to avoid information about COVID-19.

#### Information avoidance and health literacy

As shown in Table 3, after keeping sex, age, race/ethnicity, education, perceived risk, and worry constant, those with lower health literacy were more likely to avoid information about COVID-19. This negative association between health literacy and information avoidance was consistent across all types of health literacy measures: NVS scores (b = − 0.47, p = 0.033), eHEALS scores (b = − 0.12, p = 0.003), functional health literacy (b = − 0.66, p = 0.001), communicative health literacy (b = − 0.94, p < 0.001), information appraisal (b = − 0.36, p = 0.004), and empowerment (b = − 0.62, p = 0.027).

**Table 3:** Information avoidance and health literacy

	NVS		eHEALS		AAHLS							
	b	95% CI	b	95% CI	Functional		Communicative		Information Appraisal		Empowerment	
					b	95% CI	b	95% CI	b	95% CI	b	95% CI
Health literacy	− 0.47	[− 0.89, − 0.04]*	− 0.12	[− 0.19, − 0.04]*	− 0.66	[− 1.07, − 0.26]*	− 0.94	[− 1.27, − 0.62]**	− 0.36	[− 0.60, − 0.12]*	− 0.62	[− 1.17, − 0.07]*
Sex (ref: male)												
Female	− 0.80	[− 1.80, 0.20]	− 0.80	[− 1.79, 0.19]	− 0.83	[− 1.82, 0.16]	− 0.89	[− 1.86, 0.09]	− 0.72	[− 1.72, 0.28]	− 0.80	[− 1.79, 0.20]
Age	− 0.08	[− 0.15, − 0.01]*	− 0.07	[− 0.14, 0.00]	− 0.07	[− 0.14, 0.00]	− 0.07	[− 0.13, 0.00]	− 0.07	[− 0.14, 0.00]	− 0.08	[− 0.14, − 0.00]*
Race/ethnicity (ref: white)												
Hispanic/Latino	0.38	[− 1.36, 2.12]	0.64	[− 1.08, 2.37]	0.50	[− 1.22, 2.23]	0.46	[− 1.23, 2.15]	0.40	[− 1.33, 2.13]	0.62	[− 1.11, 2.35]
Black or African American	− 0.27	[− 2.25, 1.71]	0.17	[− 1.78, 2.12]	0.16	[− 1.79, 2.11]	0.21	[− 1.70, 2.12]	0.24	[− 1.72, 2.20]	0.09	[− 1.86, 2.05]
American Indian or Alaska Native	− 0.68	[− 2.68, 1.32]	− 0.36	[− 2.33, 1.62]	− 0.46	[− 2.43, 1.52]	− 0.38	[− 2.31, 1.55]	− 0.33	[− 2.31, 1.64]	− 0.33	[− 2.31, 1.65]
Asian	− 0.26	[− 1.96, 1.44]	0.20	[− 1.46, 1.85]	− 0.01	[− 1.67, 1.64]	0.16	[− 1.45, 1.78]	0.10	[− 1.55, 1.75]	0.19	[− 1.47, 1.85]
Other	2.70	[− 0.13, 5.52]	2.88	[0.07, 5.69]*	2.81	[0.01, 5.61]*	2.67	[− 0.08, 5.42]	2.97	[0.17, 5.78]*	2.97	[0.16, 5.79]*
Education (ref: undergraduate)												
Graduate	− 0.23	[− 1.34, 0.88]	− 0.13	[− 1.24, 0.98]	− 0.17	[− 1.27, 0.94]	− 0.19	[− 1.28, 0.89]	− 0.13	[− 1.24, 0.98]	− 0.28	[− 1.39, 0.83]
Perceived risk	− 0.35	[− 0.81, 0.10]	− 0.38	[− 0.83, 0.08]	− 0.36	[− 0.81, 0.10]	− 0.33	[− 0.78, 0.11]	− 0.33	[− 0.78, 0.13]	− 0.39	[− 0.84, 0.07]
Worry	− 1.06	[− 1.47, − 0.64]	− 1.09	[− 1.50, − 0.67]**	− 1.14	[− 1.55, − 0.72]**	− 1.10	[− 1.51, − 0.70]**	− 1.03	[− 1.44, − 0.61]**	− 0.97	[− 1.39, − 0.55]**
Adjusted R^2^	0.08		0.09		0.09		0.13		0.09		0.08	

*CI* confidence interval

^*^p < 0.05, **p < 0.001

Also, compared to older people, younger individuals were more likely to avoid COVID-19 information, when having health literacy, sex, age, race/ethnicity, education, perceived risk, and worry constant. Compared to White respondents, those who self-identified as “other” race/ethnicity were more likely to avoid COVID-19 information, when having health literacy, sex, age, race/ethnicity, education, perceived risk, and worry constant.

### Discussion

Our study contributes to the current literature by investigating the relationship between health literacy and information avoidance during the COVID-19 pandemic in the U.S. Also, we assessed the complex health literacy concept by conducting multiple measures to capture individual’s self-reported, perception based health literacy and objective, performance-based health literacy. We found that those with lower health literacy were more likely to avoid information about COVID-19. Our findings are in line with a prior study in Germany reported that lower eHealth literacy was associated with higher COVID-19 information avoidance [4]. Our study examined the health literacy concept beyond eHealth literacy and confirmed that the negative association between health literacy and information avoidance was consistent across all types of health literacy measures.

Our results indicated that the double burden of low health literacy and high information avoidance is likely to lead to a lack of knowledge about COVID-19. According to Miller’s monitoring and blunting hypothesis [22], those refusing to actively seek health information have minimum information about that health topic [23]. For example, due to high information avoidance, individuals with low health literacy might not learn about the most important preventive behaviors and the value of vaccinations to protect themselves from contagion. They might also have difficulty estimating contagion risk. To control the spread of COVID-19, it is essential to identify the groups who have the double burden of low levels of health literacy and levels of high information avoidance, as well as create strategic communication interventions to promote preventive behaviors among these individuals [24, 25].

People are more likely to avoid information if they perceive limited coping resources [1]. Coping resources include receiving social support [26], stability in other life domains [1], and having a greater number of self-aspects such as social roles, activities, and goals [27]. Thus, interventions improving coping resources could reduce information avoidance among vulnerable individuals. Furthermore, people are less likely to avoid health information if they can easily obtain and interpret the information [1]. Therefore, disseminating high-quality COVID-19 message using simple language to provide easy-to-understand information might be another effective strategy to reduce information avoidance among individuals with lower levels of health literacy.

We also found that higher worry or perceived risk did not trigger COVID-19 information avoidance; instead, those who were more worried about becoming infected with the virus and believed that they had a higher chance of infection tended to seek more information about it. Our findings align with previous studies reporting that people are less likely to avoid health information if they have higher perceived risk and/or more worry about getting the disease [4, 18–21].

The key finding from this study that low health literacy is related to health information avoidance (which can limit understanding about health risks and compliance with health promotion recommendations during a pandemic) suggests the need to develop new health communication strategies for addressing these problems. Weick’s model of organizing [28, 29] asserts that health care consumers and providers face tremendous information challenges when attempting to prevent or reduce health risks [30]. The double-problem of low health literacy and information avoidance limits vulnerable population’s access to relevant health information needed to respond effectively to the COVID-19 pandemic. According to Kreps [30], applications from Weick’s model of organizing suggest that public health officials should develop targeted health education programs for low literacy level populations that provide these groups with easy to understand and relevant pandemic-related health information to overcome their reluctance to seek health information. These targeted health communication efforts can have a profound influence on improving health outcomes.

## Limitations

The cross-sectional study design inhibits our ability to infer causal relationships between health literacy and information avoidance. Future studies can build upon this research by using a repeated measure design to evaluate the effects of information avoidance over time, especially during different times of health crises. Also, the findings might have a limited generalizability because our participants were recruited from a single university. All the participants were receiving college education and most of them were in young age. Future research might build upon this study by examining other populations, especially those who tend to have the greatest inequities in access to health information (e.g., elderly people, immigrants with limited English proficiency, and racial/ethnic minorities). In addition, future research can examine the influences of health literacy and information avoidance on important health behaviors (e.g., following prevention guidelines and receiving vaccinations) and important health outcomes (e.g., rates of infection, hospitalization, morbidity, and mortality).

## Data Availability

The dataset analyzed during the current study are not publicly available due to other manuscripts using this dataset are under preparation but are available from the corresponding author on reasonable request.

## Abbreviations

NVS: Newest Vital Sign
eHEALS: eHealth Literacy Scale
AAHLS: All Aspects of Health Literacy Scale
